# Fluoxetine and Thrombocytopenia in Bipolar Disorder: Unveiling a Rare Adverse Effect

**DOI:** 10.7759/cureus.81392

**Published:** 2025-03-28

**Authors:** Joman S Alshereida, Layth B Kinani, Mazin A Mukhtar, Samah M Qaoud

**Affiliations:** 1 Psychiatry, Al Amal Psychiatric Hospital, Emirates Health Services, Dubai, ARE

**Keywords:** bipolar disorder, fluoxetine, psychiatry, ssri, thrombocytopenia

## Abstract

Fluoxetine, a selective serotonin reuptake inhibitor (SSRI), is widely prescribed to treat depressive episodes but is rarely associated with hematological side effects such as thrombocytopenia. This report presents the case of a 26-year-old Omani man with bipolar disorder and chronic idiopathic thrombocytopenia who developed a significant decline in platelet count while being treated with fluoxetine during a depressive episode.

Thrombocytopenia, defined as a platelet count below 150 × 10³/µL, can result from various factors, including immune dysregulation, infections, or drug-induced effects. Prior to initiating fluoxetine, the patient's platelet levels had been stable. However, it progressively declined during treatment, eventually reaching a critical level of 34 × 10³/µL. Extensive investigations ruled out other causes, implicating fluoxetine as the primary contributor. Discontinuing the medication led to a gradual improvement in the patient's platelet count.

This case underscores the importance of promptly recognizing platelet decline and the need for healthcare providers to remain vigilant about SSRI-induced hematological side effects, especially in patients with pre-existing thrombocytopenia.

## Introduction

Thrombocytopenia, defined as a platelet count below 150 × 10³/µL, is a condition that can arise from a variety of underlying causes, including autoimmune diseases, infections, and medication side effects [1]. Platelets play a crucial role in hemostasis, and a significant reduction in their count can lead to an increased risk of bleeding, ranging from mild bruising to severe hemorrhage [2]. Drug-induced thrombocytopenia (DITP) is a well-documented but less common cause [3]. DITP can occur through immune-mediated mechanisms, where drug-dependent antibodies bind to platelets, leading to their destruction, or through direct toxicity to megakaryocytes, the precursor cells of platelets [4].

Selective serotonin reuptake inhibitors (SSRIs) are among the most commonly prescribed antidepressants worldwide, primarily due to their efficacy in treating depressive and anxiety disorders, as well as their relatively favorable side effect profile compared to older antidepressants [5]. However, SSRIs have been associated with various hematological side effects, including an increased risk of bleeding and, in rare cases, thrombocytopenia [6]. The mechanism by which SSRIs affect platelet function is thought to be related to their inhibition of serotonin reuptake. Serotonin is stored in platelet-dense granules and is released upon platelet activation, playing a key role in platelet aggregation [7]. By depleting serotonin stores, SSRIs can impair platelet function, potentially leading to bleeding tendencies or, rarely, thrombocytopenia [8].

Fluoxetine, one of the most widely used SSRIs, has been implicated in several case reports of thrombocytopenia, although the incidence remains low [9]. Given the widespread use of fluoxetine and other SSRIs, clinicians must be aware of this rare but potentially serious adverse effect, particularly in patients with pre-existing platelet disorders or those who are otherwise at increased risk of bleeding [6].

This case report presents fluoxetine-induced thrombocytopenia in a patient with bipolar disorder and chronic idiopathic thrombocytopenia, highlighting the importance of monitoring platelet counts during SSRI therapy, especially in at-risk patients.

## Case presentation

The present report describes a 26-year-old male patient of Omani origin with a longstanding history of bipolar affective disorder, managed primarily with mood stabilizers and antipsychotics, including lithium, haloperidol, and quetiapine. His medical history is significant for chronic thrombocytopenia, with baseline platelet levels typically ranging from 90 to 118 × 10³/µL. The patient does not have a family history of thrombocytopenia or other hematological disorders. He has a history of multiple hospitalizations for psychiatric stabilization and a prior suicide attempt by medication overdose.

The patient was admitted to a psychiatric facility in October 2023 with severe depressive symptoms, including feelings of guilt and worthlessness, and chronic suicidal ideation without a clear plan. His last suicidal attempt was within a week of admission. He denied experiencing auditory hallucinations or manic symptoms during the current episode. The patient had not been prescribed any SSRIs before. 

**Figure 1:**
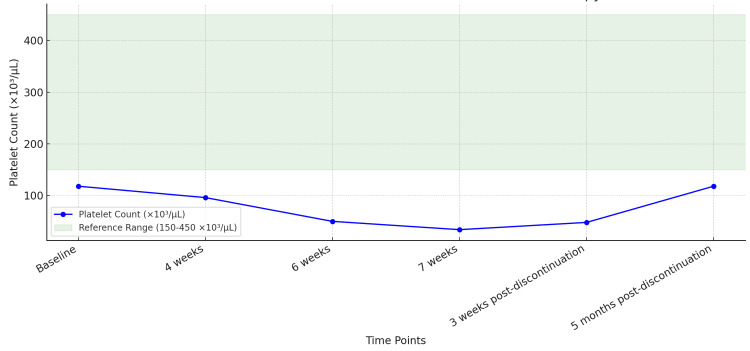
Platelet Count Over Time in Relation to Fluoxetine Therapy

To address the depressive symptoms, fluoxetine was initiated at 20 mg daily and titrated to 60 mg daily over six weeks. During this period, the patient's depressive symptoms improved marginally. However, serial monitoring of platelet levels revealed a progressive decline following fluoxetine initiation. The platelet count, which was 118 × 10³/µL at baseline, dropped to 96 × 10³/µL after four weeks. By six weeks, it further decreased to 50 × 10³/µL, eventually reaching a critical level of 34 × 10³/µL at seven weeks. This is represented in Figure 1. 

The patient's platelet count continued to decline despite stable vital signs and an absence of active bleeding or bruising. When asked, he denied experiencing any recent significant changes in lifestyle, diet, or stressors that might have influenced platelet levels at the time. The patient's medications were reviewed with a hematologist, and fluoxetine was identified as the likely cause, given the temporal association with platelet reduction.

An extensive workup was conducted to rule out alternative causes of thrombocytopenia. A hematological evaluation revealed normal white blood cell count and hemoglobin levels, with no evidence of hemolysis or abnormal red cell morphology. A bone marrow biopsy was not indicated due to known chronic thrombocytopenia. Autoimmune markers were negative for antinuclear antibodies (ANA) and other markers of autoimmune disease. Infectious workup, including HIV antibodies and p24 antigen, hepatitis B surface antigen (HBsAg), and hepatitis C antibodies (anti-HCV), was all non-reactive. Fluoxetine was the only newly introduced medication, implicating it as the likely cause of thrombocytopenia.

Given the strong temporal association and absence of other causative factors, fluoxetine was tapered but had to be stopped immediately afterward within one week, due to reaching close to critical levels of platelet counts. Platelet levels began to recover following cessation. Three weeks post-discontinuation, the platelet count was 48 × 10³/µL. Five months post-discontinuation, it returned to 118 × 10³/µL (Figure 1 and Table 1).

**Table 1:** Effect of Fluoxetine on Platelet Count, White Blood Cell Count, and Hemoglobin Levels Over Time Reference Ranges: Platelet Count, 150-450 × 10³/µL; WBC, 4.0-11.0 × 10³/µL; hemoglobin (Hb), 13.0-17.5 g/dL (males)

Week	Fluoxetine Dose (mg)	Platelet Count (×10³/µL)	WBC (×10³/µL)	Hb (g/dL)
Baseline (Week 0)	0	118	6.5	16.0
Week 4	40	96	6.3	15.8
Week 6	60	50	6.1	15.5
Week 7	60	34	6.0	15.3
3 Weeks Post-Discontinuation	0	48	6.4	16.0
5 Weeks Post-Discontinuation	0	118	6.6	16.5

To manage depressive symptoms, citalopram, another SSRI with a lower risk of hematological side effects, was initiated at 20 mg daily. The patient's mood remained stable, and no further declines in platelet count were observed.

## Discussion

The patient’s platelet count declined from 118 × 10³/µL to 34 × 10³/µL over seven weeks of fluoxetine therapy, despite stable vital signs and the absence of active bleeding or bruising. This progression aligns with findings from other case reports, suggesting a direct association between fluoxetine and thrombocytopenia. For instance, Yucel et al. reported three cases where fluoxetine was associated with thrombocytopenia, with platelet counts decreasing significantly during treatment and recovering upon discontinuation of the drug, while clearly mentioning that none of the patients had other comorbidities or lifestyle changes that could have contributed to platelet count decline [9]. Similarly, a study by Alderman et al. documented a 49-year-old man who developed a release-type defect in platelet aggregation during fluoxetine treatment, highlighting the drug's potential impact on platelet function [8].

While the exact mechanism remains unclear, fluoxetine-induced thrombocytopenia is hypothesized to involve immune-mediated platelet destruction or direct suppression of platelet production. In this case, the temporal relationship between fluoxetine initiation and the decline in platelet count, along with the absence of other causative factors, strongly implicates fluoxetine as the primary contributor. This is consistent with findings from Mirsal et al., who reported a case of ecchymosis and thrombocytopenia in a patient taking fluoxetine, which resolved after discontinuation of the drug [10]. Additionally, Fountoulakis et al. described a case where fluoxetine was associated with ecchymosis, further highlighting the drug’s potential to cause hematological side effects through mechanisms that may not always involve thrombocytopenia [11].

The patient’s platelet count began to recover upon discontinuation of fluoxetine, reaching 118 × 10³/µL five months later. This response is consistent with the reversible nature of fluoxetine-induced thrombocytopenia observed in other cases. Yucel et al. reported that platelet counts returned to normal within weeks of discontinuing fluoxetine in their cases [9]. Furthermore, Andrade and Sharma highlighted that while SSRI-induced bleeding risks are generally low, thrombocytopenia can be a serious complication, underscoring the need for prompt discontinuation of the offending drug when thrombocytopenia is detected [6].

To further assess this association, the Naranjo Adverse Drug Reaction Probability Scale was applied, yielding a total score of 8, classifying this reaction as “probable.” This assessment strengthens the argument that fluoxetine was the primary factor contributing to thrombocytopenia in this case [12].

In addition to thrombocytopenia, fluoxetine has been associated with ecchymosis and other bleeding-related complications. Akbulut et al. reported a case of breast ecchymosis in a patient taking fluoxetine, which resolved after discontinuation of the drug [13]. Similarly, Eray and Murat described an adolescent girl with trichotillomania who developed ecchymosis while on fluoxetine, further emphasizing the drug’s potential to cause bleeding complications even in younger populations [14]. These cases suggest that fluoxetine’s impact on platelet function may manifest as ecchymosis or other bleeding-related symptoms, even in the absence of severe thrombocytopenia.

Beyond fluoxetine, other antidepressants have also been implicated in thrombocytopenia. For instance, paroxetine, an SSRI with potent anticholinergic properties, has been associated with isolated cases of thrombocytopenia [15]. Notably, venlafaxine, a serotonin-norepinephrine reuptake inhibitor (SNRI), was also linked to thrombocytopenia in a 71-year-old patient, though such reports remain rare [16].

In this case, the decision to discontinue fluoxetine and switch to citalopram, another SSRI with a lower risk of hematological side effects, proved effective. The patient’s mood remained stable, and no further declines in platelet count were observed. This response is consistent with the reversible nature of fluoxetine-induced thrombocytopenia observed in other cases. Laporte et al. conducted a meta-analysis of observational studies and found that SSRIs, including fluoxetine, are associated with an increased risk of bleeding, particularly in patients with additional risk factors such as older age or concomitant use of anticoagulants [17]. This further supports the need for careful monitoring and individualized treatment decisions in patients receiving SSRIs.

This case underscores the importance of monitoring platelet counts in patients initiating SSRI therapy, especially those with pre-existing thrombocytopenia or platelet disorders. While the absolute risk of significant bleeding is low, clinicians should remain vigilant for signs of bleeding, such as ecchymosis, and consider alternative antidepressant therapies if thrombocytopenia develops. The patient's recovery upon discontinuation of fluoxetine supports the need for prompt recognition and management of this adverse effect.

## Conclusions

Fluoxetine-induced thrombocytopenia, though rare, is a significant concern, particularly in patients with underlying platelet abnormalities. This case highlights the necessity for healthcare providers to be aware of this potential side effect and the need for routine platelet count monitoring in patients with pre-existing thrombocytopenia who are prescribed SSRIs to ensure patient safety. A multidisciplinary approach involving psychiatrists and hematologists could also be considered. Further research is warranted to establish standardized guidelines for SSRI-induced thrombocytopenia. 
